# Computational Fluid Dynamics in Medicine and Biology

**DOI:** 10.3390/bioengineering11111168

**Published:** 2024-11-20

**Authors:** Amirtahà Taebi

**Affiliations:** Biomedical Engineering Program, Mississippi State University, 130 Creelman Street, Mississippi State, MS 39762, USA; ataebi@abe.msstate.edu

## 1. Introduction

This Special Issue of *Bioengineering* presents cutting-edge research on the applications of computational fluid dynamics (CFD) in medical and biological contexts. The papers in this collection showcase how CFD can advance our understanding of complex physiological phenomena and improve treatment strategies across various fields of biomedicine.

## 2. Exploring the Flows of Life: CFD in Medicine and Biology

Beginning with the heart, Das et al. use both CFD simulations and a physical mock flow loop to investigate a novel surgical procedure, the Hybrid Comprehensive Stage II (HCSII), for treating single ventricle heart anomalies [1]. This approach enables a thorough evaluation of the procedure’s effectiveness, showcasing how CFD can assist in designing and validating innovative surgical techniques. Addressing myocardial perfusion, Pannone proposes a theoretical fluid mechanical model in both healthy and stenotic conditions [2]. This model, based on Terzaghi’s consolidation theory, simulates the swelling and drainage cycles of the heart to analyze blood pressure and flow rate across the ventricle wall. The findings show that this model can effectively reproduce the mechanisms of healthy and ischemic perfusion, providing insights into the hemodynamics of heart diseases.

Moving from the heart to the aorta, Schwartz de Azevedo et al. address the prediction of ascending aortic aneurysm growth in a longitudinal study [3]. Their study leverages patient-specific aortic models and CFD simulations to reveal how variations in aortic geometry influence the development of these potentially fatal bulges. By pinpointing hemodynamic factors associated with aneurysm growth, this research provides valuable insights for risk assessment and treatment planning. Similarly, Albadawi et al. investigate the impact of arterial wall compliance on blood flow dynamics in stenotic coronary and carotid arteries [4]. They use CFD to simulate blood flow in both elastic and rigid models of coronary and carotid arteries, finding that while a rigid wall assumption may be sufficient for larger arteries like the carotid, considering wall elasticity is essential for accurate modeling of smaller arteries.

CFD also plays a crucial role in understanding the blood flow in the cerebral vessels. Uchiyama et al. use simulations to compare the effectiveness of different flow diverter configurations for treating cerebral aneurysms [5]. Their findings indicate that overlapping flow diverters significantly reduce blood flow velocity and wall shear stress within the aneurysm, paving the way for improved treatment outcomes.

Shifting to the microscopic scale, the study by Salman et al. employs two-dimensional CFD simulations to explore the dynamics of motile cilia in the brain [6]. These tiny hair-like structures, lining the brain’s ventricles, create currents that drive cerebrospinal fluid (CSF) flow. This research underscores the critical importance of cilia tilt angle and synchronized beating for efficient fluid transport, uncovering fundamental mechanisms governing CSF dynamics and their significance for brain health.

Li et al. explore a novel approach to nasal drug delivery, targeting the olfactory region for potential treatment of central nervous system disorders [7]. Their simulations demonstrate how magnetic fields can guide charged drug particles to precise locations within the nasal cavity. This systematic examination of particle size, injection parameters, and magnetic field strength opens up new possibilities for optimizing drug delivery efficiency and targeting specific regions.

Turova et al. present a mathematical model of an acoustic shear wave biosensor to sense the glycocalyx, a delicate layer lining blood vessels [8]. Their model investigates the interaction of acoustic waves with the glycocalyx and the surrounding fluid, offering a non-invasive method to assess glycocalyx properties and detect potential damage.

Finally, the future of CFD in medicine and biology is bright, especially with the integration of recent advances in machine learning. Machine learning can be used to accelerate CFD simulations, automate the analysis of complex flow patterns, and even develop predictive models for disease progression and treatment outcomes [9]. For instance, machine learning algorithms can be trained on large datasets of patient-specific geometries and hemodynamic data to identify subtle relationships between flow characteristics and clinical outcomes. This could lead to the development of personalized risk assessment tools and the optimization of treatment strategies for various cardiovascular diseases. Additionally, machine learning can be used to improve the accuracy and efficiency of boundary condition selection in CFD simulations, further enhancing the reliability and predictive power of these models. The synergy between CFD and machine learning holds immense potential to revolutionize the field, leading to a new era of precision medicine and personalized healthcare.

## 3. Conclusions

This collection of papers showcases the impressive versatility of CFD as a tool for both clinical applications and fundamental biological research. The studies presented here, spanning a wide range of scales and medical conditions, demonstrate the transformative potential of CFD to enhance healthcare outcomes, guide the development of medical devices, and deepen our understanding of the intricate fluid dynamics in living systems. From the macroscopic level of blood flow in arteries to the microscopic world of motile cilia and the delicate glycocalyx, CFD provides a powerful lens through which to visualize and understand the complex interplay between fluid mechanics and biological processes. As technology advances and computational power continues to grow, CFD is poised to play an even greater role in unraveling the mysteries of life and driving innovation in healthcare.
